# Assessing the collection and reporting of patient-reported outcome data in interventional cancer trials: a single institution, retrospective systematic evaluation

**DOI:** 10.1186/s41687-022-00529-9

**Published:** 2022-12-22

**Authors:** Emma Lidington, Holly Hogan, Ann Gandolfi, Jane Lawrence, Eugenie Younger, Helena Cho, Clare Peckitt, Kabir Mohammed, Sheila Matharu, Lisa Scerri, Olga Husson, Susanne Cruickshank, Rachel Turner, Linda Wedlake

**Affiliations:** 1 PROFILES Team, The Royal Marsden NHS Foundation Trust, London, UK; 2 Research & Development, The Royal Marsden NHS Foundation Trust, London, UK; 3 Sarcoma Unit, The Royal Marsden NHS Foundation Trust, London, UK; 4 Digital Services, The Royal Marsden NHS Foundation Trust, London, UK; 5 Division of Clinical Studies, Institute of Cancer Research, London, UK; 6 Applied Health Research, The Royal Marsden NHS Foundation Trust, London, UK

**Keywords:** PROs, PROMs, PREMs, Data completeness, Missing data, SPIRIT-PRO, CONSORT-PRO

## Abstract

**Background:**

To understand our performance with respect to the collection and reporting of patient-reported outcome (PRO) measure (PROM) data, we examined the protocol content, data completeness and publication of PROs from interventional trials conducted at the Royal Marsden NHS Foundation Trust (RM) and explored factors associated with data missingness and PRO publication.

**Design:**

From local records, we identified closed, intervention trials sponsored by RM that opened after 1995 and collected PROMs as primary, secondary or exploratory outcomes. Protocol data were extracted by two researchers and scored against the SPIRIT-PRO (PRO protocol content checklist; score 0–100, higher scores indicate better completeness). For studies with locally held datasets, the information team summarized for each study, PRO completion defined as the number of expected (as per protocol) PRO measurements *versus* the number of actual (i.e. completed) PRO measurements captured in the study data set. Relevant publications were identified by searching three online databases and chief investigator request. Data were extracted and each publication scored against the CONSORT-PRO (PRO manuscript content checklist; scored as SPIRIT-PRO above). Descriptive statistics are presented with exploratory comparisons of point estimates and 95% confidence intervals.

**Results:**

Twenty-six of 65 studies were included in the review. Nineteen studies had accessible datasets and 18 studies published at least one article. Fourteen studies published PRO results. Most studies had a clinical (rather than PRO) primary outcome (16/26). Across all studies, responses in respect of 35 of 69 PROMs were published. Trial protocols scored on average 46.7 (range 7.1–92.9) on the SPIRIT-PRO. Among studies with accessible data, half (10/19) had less than 25% missing measurements. Publications scored on average 80.9 (range 36–100%) on the CONSORT-PRO. Studies that published PRO results had somewhat fewer missing measurements (19% [7–32%] vs 60% [− 26 to 146%]). For individual PROMs within studies, missing measurements were lower for those that were published (17% [10–24%] vs 41% [18–63%]). Studies with higher SPIRIT-PRO scores and PROs as primary endpoints (13% [4–22%] vs 39% [10–58%]) had fewer missing measurements.

**Conclusions:**

Missing data may affect publication of PROs. Extent of inclusion of SPIRIT-PRO protocol items and PROs as primary endpoints may improve data completeness. Preliminary evidence from the study suggests a future larger study examining the relationship between PRO completion and publication is warranted.

**Supplementary Information:**

The online version contains supplementary material available at 10.1186/s41687-022-00529-9.

## Introduction

The importance of patient-reported outcomes (PROs) is increasingly recognised in cancer research [1]. PROs refer to “reports coming directly from patients about how they feel or function in relation to a health condition and its therapy without interpretation by healthcare professionals or anyone else” [2]. PROs may include concepts such as symptoms, quality of life, functional status or health and its treatment. Patient-reported outcome measures (PROMs) are tools for capturing PROs, usually in the form of questionnaires [3]. Using PROMs to investigate how medicinal and non-medicinal interventions effect patients’ lives beyond routine clinical outcomes in ways such as role functioning or social functioning provides richer information to enable patients, clinicians and commissioners to make appropriate clinical decisions.

Despite growing interest in PRO research, studies show there is room for improvement in the design, collection, analysis and reporting of PROs [4–8]. A recent review of trials that collected PRO data found a third did not include PRO-specific information in the protocol, with incomplete details in those that did [5]. Perhaps more disconcertingly, over a third of the studies in the review failed to publish the PRO results in a primary or secondary article. This raises an important ethical question, as data captured using these instruments but not reported is a waste of patient and researcher time and resource. To address deficits in the specification and reporting of PROs, PRO extension requirements have been added to the SPIRIT and CONSORT standards encouraging investigators to address critical requirements for protocol content and reporting when using PROs in clinical trials [9–11]. The checklists allow researchers to score protocols and publications to indicate the level of ‘completeness’ of PRO information.

One limitation in the Kyte et al. study was the lack of access to study datasets to explore whether the PRO data was ever initially captured. While there is recognition that missing data is a key issue in PRO research, it is unclear to what extent missing data contributes to failure to publish [12]. While funding and regulatory bodies are starting to encourage public availability of data, actual access to datasets is still lacking [13]. Even among trials that make information available, unpublished data is unlikely to be shared, limiting the ability to answer this question.

To have equal access to published and unpublished data, we explored the relationship between missing PRO data and publication by interrogating the study data sets available in the central trial database repository (CRS Web) at our institution. Specifically looking at interventional studies that planned to use PROs, our main objectives were to (1) describe our performance with respect to the completeness of PRO data collected and reported, (2) explore study-related factors associated with PRO data completeness and reporting and (3) examine the extent to which studies addressed SPIRIT-PRO and CONSORT-PRO requirements [9, 10].

## Methods

### Protocol registration

The protocol for this service evaluation was registered on Open Science Framework on 02/07/2020 (https://osf.io/ga3cn/).

### Review process

The work was handled under the framework of a UK NHS Service Evaluation. This Service Evaluation (registration number: SE920) was approved by the Institution’s independent Committee for Clinical Research (CCR) which includes representatives from The Royal Marsden NHS Foundation Trust (RM) and Institute of Cancer Research (ICR). Access to encoded data to assess PRO completion was given only with Chief Investigator consent. Only the presence of a response was assessed. The responses themselves were not examined. PRO completion was summarised by the information team and exported for analysis to a trusted research environment with access limited to the Scoping Review Team. Access to withdrawal information was permitted in order to calculate the expected number of PRO measurements.

### Study inclusion

Eligible studies included closed interventional studies sponsored by RM that intended to collect PROs. Using EDGE, the institution’s clinical research management system, we identified potential studies from the start of local digital records (1995). The search terms used to interrogate EDGE were ‘RM Sponsor’ AND ‘Interventional’ AND ‘Closed’. Interventions included clinical trials of investigative medicinal products (CTIMPs) and non-CTIMPs. Available trial protocols for all studies satisfying these criteria were provided by the Research and Development Department and screened for use of PROMs or patient reported experience measures (PREMs). Studies not intending to use PROMs or PREMs were excluded from the review. Included studies were assigned a random ID from 1 to 26.

### Protocols: data extraction and SPIRIT-PRO scoring

Two reviewers (EL and HH) independently screened each protocol to extract relevant data using a pre-defined data extraction table (Additional file 1: Table S1) and scored each protocol using the SPIRIT-PRO checklist [10]. A third reviewer (LW) arbitrated discrepancies between reviewers. Data extracted included study characteristics, inclusion/exclusion criteria, funding source (commercial, academic or hybrid) and characteristics of each PROM used by each study. Healthcare cost measures do not fit entirely within our definition of PROMs, however, we decided to include these in our review as they add burden to participants and may be at particular risk for under-reporting as they require specific expertise for analysis.

In total, 25 data items were extracted per protocol with an additional 4 PROM specific items extracted for each PROM used. For each protocol, all 16 SPIRIT-PRO checklist items were recorded as ‘present’, ‘absent’ or ‘not applicable’. Percent agreement was calculated between reviewers as a measure of inter-rater reliability. A total SPIRIT-PRO score ranging from 0 to 100 was calculated for each study as the number of checklist items present divided by the sum of items present and absent (present/(present + absent)). This allowed us to adjust the denominator where checklist items were deemed ‘not applicable’. Summary tables were compiled for study characteristics, PROM characteristics and SPIRIT-PRO scores.

### Trial data: acquisition and aggregation

For all included studies, permission from the Chief Investigator (CI) was sought to access anonymized trial data sets held on the Institution’s centralised trial database platform. Where trial data sets were not available from the central platform, the CI shared the PRO data directly with the information team.

On receipt of CI permission, the Information team summarized the completeness of the data for each PROM in each study to provide researchers with an aggregate dataset for analysis. This PROM-level information included the estimated number of expected and missing ‘measurements’, defined here as the unique completion of a PROM by each participant at each timepoint, and (among present measurements) the number of expected and missing items. Expected and missing measurements for each PROM were estimated by multiplying the number of timepoints expected (as detailed in the protocol schedule of assessments) by the number of participants in the study. The expected number was reduced with early withdrawal due death or study exit. Where available, study publications were examined to verify number of withdrawals. Expected items for each PROM were equivalent to the total number of items in the PROM multiplied by the number of available measurements. Missing items were the total number of missing items for the PROM in the data sets across all measurements. The number missing was then divided by the number expected to get percent missing for both measurements and items.

The Information team then imported the aggregate-level PRO completion data sets for each study into BRIDgE, the Institution’s trusted research environment. Access to BRIDgE was password protected and limited to the Scoping Review team, who combined the PRO completion data with protocol and publication data sets for analysis.

### Publications: identification, data extraction and CONSORT-PRO scoring

To identify all available published articles, researchers agreed search terms for interrogation of online databases a-priori (available in the protocol). Literature searches were then undertaken independently by two researchers for each study using Medline, Embase and PsycINFO. Full articles of all trial-related primary and secondary publications were obtained. Where articles were not readily identifiable or available via online sources, the CI was contacted to determine whether an article had been published. All available publications were independently screened by two researchers and relevant data extracted using a pre-defined data extraction table (Additional file 2: Table S2). Data extracted included publication characteristics, sample demographics and PRO results. Discrepancies between reviewers were arbitrated by a third reviewer.

Articles that reported PRO results were then scored by two independent reviewers (EL and LW) using the CONSORT-PRO checklist [10]. For each article all 14 CONSORT-PRO checklist items were recorded as ‘present’, ‘absent’ or ‘not applicable’. The total score ranging from 0 to 100 was calculated using the same method as the SPIRIT-PRO above. Percent agreement between reviewers was calculated.

### Statistical methods

The aggregate-level, PRO completion data for each study was linked with the protocol and publication data sets in BRIDgE and analysed using ‘R’ (version 4.04). Descriptive statistics were calculated using frequency and percentage for categorical data or mean and standard deviation for continuous data. Absent SPIRIT-PRO and CONSORT-PRO criteria are described. The review was not formally powered and due to relatively small number of studies included, statistical analysis was limited. However, we present selected exploratory comparisons with point estimates and 95% confidence intervals (CIs) using the t-distribution. Confidence intervals for proportions were calculated using Wilson intervals. We first analyzed the data at study-level and then, to further investigate variation in missingness and publication, we analyzed the data for each PROM across all studies, referred to as ‘PROM-level’ analysis.

## Results

**Fig. 1:**
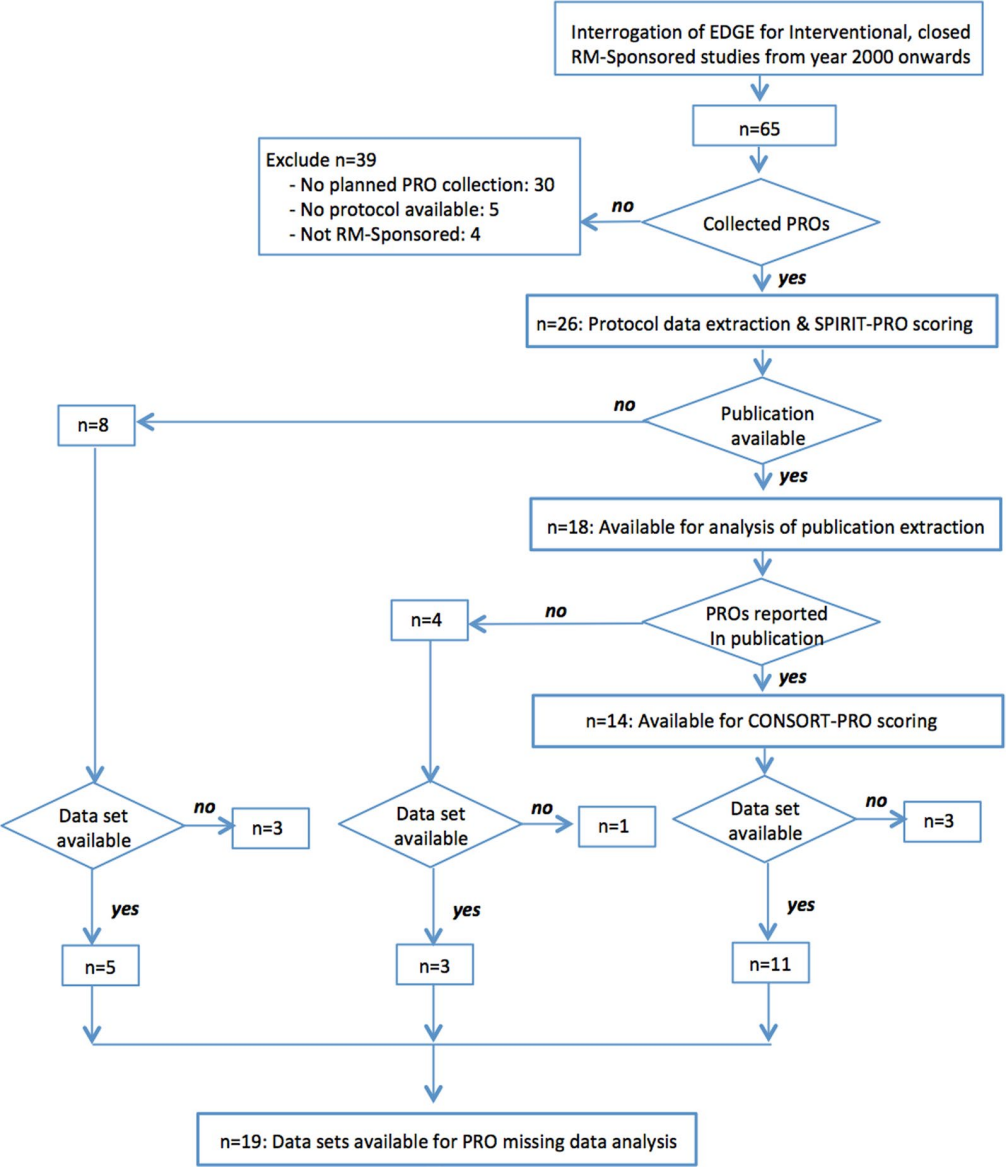
CONSORT diagram: data flow

Interrogation of EDGE revealed 65 closed, interventional, RM-sponsored studies (Fig. 1). Of these, 26 (40.0%) studies planned to collect PRO data and were eligible for inclusion. Datasets were accessible for 19 studies and publications were identified for 18 studies, of which 14 published PRO results. In total, 11 studies had all information (i.e. protocol, publication and aggregate PRO completion data sets) available.

### Protocols

*Study characteristics*: Studies included participants with a range of cancer types with the largest proportion (7/26) recruiting participants with cancers of mixed origin (Table 1). In ten studies (38.5%), the primary outcome was a PRO. Four of these were in the palliative care setting, two for treatment of lung cancer, one in the intensive care setting, one in complementary therapies, one examining hyperbaric therapy and one in gastrointestinal cancers. A wide range of interventions were evaluated ranging from device trials (n = 3), complimentary therapy (n = 3), anti-cancer systemic therapy (n = 4) and dietary interventions (n = 2). The majority (19/26) were randomised controlled trials by design, academically funded (19/26) and single-centre studies (17/26). The median (range) year of trial start was 2010 (2000–2015) and closure was 2014 (2011–2018). The average number of studies using PROs increased over time from 1.2 studies per year between 2000 and 2007 to 2.7 studies per year between 2008 and 2015. However, the proportion of studies using PROs decreased over the same time periods from 34 to 23%. Median planned sample size was 84 (25–730). The combined target recruitment for the 26 included studies amounted to 3157 participants.

**Table 1:** Baseline study characteristics (n = 26)

Study characteristics	No. of trials (%)
*Cancer type*	
Mixed	7 (27)
Head and neck cancer	5 (19)
Prostate cancer	3 (12)
Lung cancer	3 (12)
Breast cancer	3 (12)
Rectal cancer	2 (8)
Oesopho-gastric cancer	1 (4)
Pancreatic cancer	1 (4)
Pelvic cancer	1 (4)
*Primary outcome*	
Clinical endpoint	16 (62)
Patient-reported endpoint	10 (39)
*Intervention*	
Systemic therapy	4 (15)
Symptom control	4 (15)
Radiotherapy	3 (12)
Medical device	3 (12)
Complementary therapy	3 (12)
Patient-directed therapy	3 (12)
Dietary	2 (8)
Analgesic	1 (4)
Diagnostic	1 (4)
Supportive care	1 (4)
Bariatric therapy	1 (4)
*Trial design*	
RCT	19 (73)
Single-arm trial	5 (19)
Randomised cross-over trial	2 (8)
*Number of centres*	
Singe-centre	17 (65)
Multi-centre	9 (35)
*Funding*	
Academic	19 (73)
Industry	3 (12)
Hybrid	2 (8)
Unknown	2 (8)
*Planned sample size*	
< 100	15 (58)
100–199	8 (31)
> 200	3 (12)
*Sample size reached (N* = 22)*	
Yes	13 (59)
No	9 (41)

RCT, randomised controlled trial

*Unknown for 4 studies as no publication or dataset available

*PRO characteristics*: A total of 60 PROMs (49 unique instruments) were used across the 26 studies with a combined total of 154 time points resulting in a planned 41,736 PROM measurements (Table 2). The most commonly used measure was the European Organisation for Research and Treatment of Cancer (EORTC) Quality of Life Core Module (QLQ-C30) (10 trials; 39%). Where cancer site-specific EORTC modules were used, these were always used in conjunction with the QLQ-C30 (as recommended). The vast majority of PROMs used (36/49; 74%) were validated. Non-validated questionnaires measured healthcare costs, tolerability, toxicity, sleep and patient experience. The median number of PROMs employed was 2 and ranged from 1 (7/26 trials) to 8 (1/26 trials) with the majority of trials (10/26) using 2 PROMs (Table 3). The most commonly measured PRO concept was quality of life (58% of trials) followed by symptoms (35%) and patient experience (31%). Other concepts reported included anxiety / depression, health status, economics, well-being and physical function (Table 3).

**Table 2:** List of PRO measures used (n = 26)

Patient reported outcome title	No. of trials (%)
EORTC-QLQ-C30	10 (39)
Patient experience questionnaires (various, non-validated)	6 (23)
+ Visual analogue scales (various, validated)	5 (19)
EORTC-QLQ-LC13	3 (12)
Hospital Anxiety and Depression Scale	3 (12)
EQ-5D	3 (12)
Economics questionnaires (various, non-validated)	3 (12)
EORTC-QLQ-HN35	2 (8)
+ Brief Pain Inventory	2 (8)
+ Inflammatory Bowel Disease Questionnaire	2 (8)
Medical Research Council Dyspnoea Scale	2 (8)
St George’s Respiratory Questionnaire	2 (8)
UCLA Prostate Cancer Index	1 (4)
Functional Assessment of Cancer Therapy General Questionnaire	1 (4)
Xerostomia Questionnaire	1 (4)
EORTC-QLQ-CR29	1 (4)
Cleveland Clinic Incontinence Score	1 (4)
*Toxicity questionnaire (non-validated)	1 (4)
Borg Dyspnoea Scale	1 (4)
Lar Scales	1 (4)
EORTC-QLQ-PAN26	1 (4)
EORTC-QLQ-CR38	1 (4)
*Numerical Rating Scale	1 (4)
Neuropathic Pain Symptom Inventory	1 (4)
Short Form-36	1 (4)
Appetite, Hunger and Sensory Perception Questionnaire	1 (4)
University of Washington Quality of Life Questionnaire	1 (4)
*Measure Yourself Concerns and Wellbeing Questionnaire	1 (4)
Leeds Assessment of Neuropathic Symptoms and Signs Pain Score	1 (4)
*Palliative care Outcome Scale	1 (4)
Dyspnea-12 Scale	1 (4)
King’s Brief Interstitial Lung Disease Questionnaire	1 (4)
Expanded Prostate Cancer Index Composite	1 (4)
Tolerability (non-validated)	1 (4)
Body Image Scale	1 (4)
*Richards-Campbell Sleep Questionnaire	1 (4)
Sleep in the ICU questionnaire (non-validated)	1 (4)

EORTC, European Organisation for Research and Treatment of Cancer; QLQ, quality of life questionnaire; C30, core module 30 items; LC13, lung cancer module 13 items; EQ-5D, Euroqol questionnaire 5 dimensions; HN35, head and neck cancer module 35 items; UCLA, University of California, Los Angeles; CR29, colorectal cancer module 29 items; PAN26, pancreatic cancer module 26 items; CR38, colorectal cancer module 38 items; ICU, intensive care unit

*Used as primary outcome measure for one study; + used as primary outcome measure for two studies

**Table 3:** Number of PROs used per trial and PRO concepts measured

Variable	No. of trials (%)
*Number of PROMs used*	
1	7 (27)
2	10 (39)
3	2 (8)
4	3 (12)
5	2 (8)
6	1 (4)
8	1 (4)
*PRO concept measured*	
Quality of life	15 (58)
Symptom(s)	9 (35)
Patient experience	8 (31)
Toxicity	3 (12)
Anxiety/depression	3 (12)
Health status	3 (12)
Economics	3 (12)
Well-being	3 (12)
Physical function	2 (8)

PRO, patient-reported outcome; PROM, patient-reported outcome measure

**Fig. 2:**
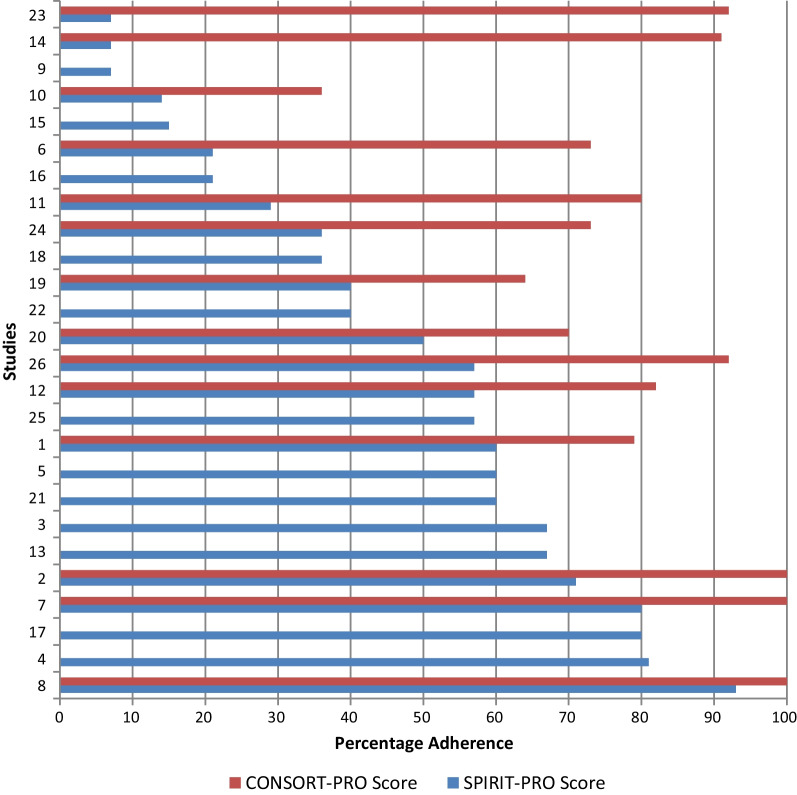
SPIRIT-PRO and CONSORT-PRO scores by study. Key: SPIRIT-PRO scores (n = 26); CONSORT-PRO scores (n = 14)

*SPIRIT-PRO scores* Trial protocols scored an average of 46.7 (range 7.1–92.9) on the SPIRIT-PRO checklist (Fig. 2). Reviewers achieved 81% agreement. Protocols included an average of 7 (range 1–13) of the 16 checklist items (Fig. 2). SPIRIT-PRO recommendations most frequently absent included item 10 (availability of multiple language versions of PROMs) and 16 (real-time monitoring of PRO responses) each omitted in 25 protocols; item 1 (responsibility for PRO content of protocol) absent in 21 protocols and item 15 (handling of missing data) absent in 19 protocols. Items 11 (inclusion of proxy reported outcomes) and 4 (PRO specific eligibility criteria) were deemed ‘not applicable’ in 25 and 15 protocols respectively. Trials obtaining the five highest SPIRIT-PRO scores were for trials evaluating supportive care (n = 1), complementary therapy (n = 1), dietary (n = 1), bariatric therapy (n = 1) and systemic treatment (n = 1) interventions.

### Trial data

Aggregate PRO completion data sets were available for 73% (19/26) of eligible studies. At the study-level, 53% (10/19) of studies had less than 25% missing measurements, 37% (7/19) of studies had between 26 and 50% missing measurements and 11% (2/19) of studies had more than 50% missing measurements. Among the 19 studies with data available, 24,402 measurements were collected of the 33,006 planned (74%). Data were accessible for three of the four studies that published a paper without publishing PRO results. Missing measurements for these studies ranged from 39 to 100%.

At the PROM-level, the studies planned to collect 69 PROMs. Data were available for 54 PROMs across the 19 studies with accessible data sets. Sixty-three percent of PROMs (34/54) had 25% or fewer missing measurements, 22% of PROMs (12/54) had between 26 and 50% missing measurements and 15% of PROMs (8/54) had greater than 50% missing measurements. Among available measurements with more than one item (n = 46), 83% of PROMs (45/46) had 25% or fewer missing items and only one PROM (1/46) had between 26 and 50% missing items.

### Publications

*Article characteristics* Publications were available for 18 (69%) of the 26 eligible studies, of which 78% (14/18) published results for at least one PRO. Overall, of the 26 studies intending to collect PRO data using 69 different PROMS, fourteen studies collectively published 35 (51%) of the intended PROMs. Of those that published PROs, the median time between study closure and publication of PRO results was 2 years (range − 1 to 3). Median year of closure for studies without an identified publication (8/26) was 2016 (range 2011–2018). Information about the final sample size was found in either the data or publication for 23 studies and indicated 61% (14/23) of studies achieved the planned sample size.

*CONSORT-PRO scores* Publications containing PRO results were available for 14 studies. Where more than one publication reported PRO results for a study, the primary publication was scored. An average of 9 (range 4–13) of the 14 CONSORT-PRO checklist items were present in each publication. Overall, publications scored an average of 80.9 (range 36.4–100.0) (Fig. 2). Reviewers achieved 77% agreement. CONSORT-PRO recommendations most frequently absent included item 7 (approaches for dealing with missing data), which was absent in 7 publications, item 9 (baseline PRO data) absent in 5 publications and item 3 (PRO hypothesis) absent in 5 publications. Items 4 (PRO specific eligibility criteria) and 12 (additional PRO analyses) were deemed ‘not applicable’ in 12 and 10 publications respectively. Trials obtaining the five highest CONSORT-PRO scores evaluated supportive care (n = 1), complementary therapy (n = 2) and dietary (n = 2) interventions.

### Study-level analysis

Compared to studies with a primary clinical endpoint (16/26), studies with a primary PRO endpoint had higher mean [95% CI] SPIRIT-PRO scores (35.2 [23.2–47.3] vs 65.1 [51.3–78.9], respectively). Looking at studies that published at least one manuscript, there was no difference in the percentage of studies [95% CI] publishing PRO results between studies with a primary PRO endpoint (7/18) and those with a clinical endpoint (86% [49–97%] vs. 73%, [43–90%], respectively). Mean [95% CI] SPIRIT-PRO scores did not differ between studies that published at least one PRO (14/18) and those that did not (44.5 [28.9–60.1] vs 32.1 [− 4.3 to 68.6], respectively).

However, among studies that published PRO results, studies with primary PRO endpoints (6/14) had slightly higher mean [95% CI] CONSORT-PRO scores (90.6 [77.9–103.3] vs 73.4 [58.5–88.3] in studies with clinical endpoints). A SPIRIT-PRO score of > 50.0 appears to consistently result in higher CONSORT-PRO scores (Fig. 2).

Focusing on studies with PRO completion data sets available, those with primary PRO endpoints (9/19) had lower mean [95% CI] percentage of missing measurements (13% [4–22%] vs 39% [20–57%] in studies with primary clinical endpoints). There was no difference in mean [95% CI] percentage of missing measurements between single centre (12/19) and multi-centre studies (21% [9–32%] vs 36% [7–65%], respectively). Studies with higher SPIRIT-PRO scores were likely to have fewer missing measurements as SPIRIT-PRO scores and the percent of missing measurements at the study-level were negatively correlated (n = 19; r =  − 0.53). Among studies with data that published at least one manuscript and included at least one PRO (11/14), the mean [95% CI] percentage of missing measurements was somewhat lower compared to studies that published a manuscript but did not include any PRO results (19% [7–32%] vs 60% [− 26 to 146%], respectively).

### PROM-level analysis

Studies that published at least one manuscript accounted for 51 of the 69 (74%) PROMs intended to be collected. Among studies that published, the percentage [95% CI] of PROMs used for primary outcomes (7/51) that were published was no different than those used for secondary outcomes (86% [49–97%] vs 66% [51–78%], respectively).

Summary missingness information was available for 54 PROMs. Among studies with summary information, PROMs used for primary objectives (9/54) had lower mean [95% interval] percentage of missing measurements than PROMs used for secondary objectives (7% [4–10%] vs 26% [18–33%], respectively). The mean [95% CI] percentage of missing measurements was lower among PROMs with published results (30/41) compared to non-published PROMs (17% [10–24%] vs 41% [18–63%], respectively).

## Discussion

This service evaluation sought to explore our Institution’s performance with respect to the completeness of PRO data collected and reported in intervention studies and identify research study specific factors associated PRO data missingness. Nearly half the studies included in our evaluation planned to capture PRO data reflecting the importance investigators attach to these measures. However, one third of trials have yet to publish any results 3–10 years since trial closure. Among those that published at least one paper, nearly a quarter did not include any PRO results and almost a third of questionnaires were never published.

We found the omission of PRO results in publications may be influenced by the degree of missing data. In this evaluation, the mean percentage of missing measurements was higher for PROMs that were not published compared to those that were published. A similar pattern to some extent was found at study-level. Furthermore, the three studies that published at least one paper without PRO results for which we had data all had more than 50% missing measurements. While our sample size is small, the findings suggest the extent of PRO data missingness may be a factor in non-publication.

We hypothesize that the completeness of PRO data may be related to the importance given to the PRO data in trial set up and conduct. Indeed, Kyte et al. found that protocols were more likely to be complete and results published where the primary outcome was a PRO [5]. Our results on data missingness align with these findings as the percentage of missing PRO measurements was lower among trials with a primary PRO endpoint compared to those with a primary clinical endpoint. This may reflect trial staff perception that primary endpoint data is more important than secondary endpoint data. Similarly, at PROM-level, we found that for PROMs employed as primary endpoints, the mean number of missing measurements was lower than for PROMs used for secondary objectives. To improve PRO data completeness by increasing perceived importance, investigators could include PROs as co-primary endpoints or use behaviour theory-informed motivational information in the protocol and trial staff training.

Our analysis of the 19 studies for which aggregate PRO completion data sets were available revealed that about half the trials had more than 25% missing measurements including two trials which had over 50% missingness. The reason for missing PRO data could reflect either that the required PRO data were not captured at all or that the data were captured and not transcribed into an accessible digital platform. Few trial protocols included in this review outlined the mode of PRO capture. Most will have used paper questionnaires, which can be cumbersome, resource-intensive and potentially prone to transcription error. Electronic capture of PRO data directly from patients shows promise but is yet to be tested in nested randomized trials. RM has recently implemented an electronic questionnaire system developed in the Netherlands called PROFILES [14, 15]. We hope to evaluate its effect on our performance in respect of PRO data capture and reporting in the future and are satisfied that we now have a reliable benchmark to assess our future performance and encourage improvements.

One way of improving the publication of PRO results is to improve the PRO-related content of trial protocols using the SPIRIT-PRO checklist. Previous research suggests that including SPIRIT-PRO checklist items improves the completeness of PRO information in protocols and may reduce the risk of bias [16]. Although publication of the SPIRIT-PRO extension in July 2018 post-dates the protocols included in this analysis, it is encouraging to see that adherence to the guidelines compares well with previously published results [5]. Our own analysis supports the hypothesis that higher SPIRIT-PRO scores have beneficial effects. In the 19 studies for which data sets were available for analysis, we found that higher SPIRIT-PRO scores were associated with fewer missing measurements. Investigators in future should make use of the SPIRIT-PRO in the protocol-writing phase. Based on the results of this evaluation, RM has implemented standard review of protocols against the SPIRIT-PRO as part of the sponsorship process to improve PRO data collection.

SPIRIT-PRO scores were higher in studies with primary PRO endpoints compared to those with primary clinical endpoints suggesting that a more thorough approach to protocol development is adopted when PROs are the primary endpoints. Similarly, CONSORT-PRO scores were higher amongst studies using PROs as the primary endpoint compared to those with primary clinical endpoints, which suggests rigor in planning translates into rigorous reporting. Adherence of the published articles to CONSORT-PRO criteria in our cohort was substantially higher than the results published by Kyte et al. [5].

This study is limited by the small sample size and single institution nature so all findings should be considered exploratory. Some of the very large confidence intervals reflect that the true estimate could not be determined based on the small sample. However, this preliminary evidence of a relationship between missing PRO data and publication provides rationale for a future research initiative to explore the relationship in more depth. The findings also suggest important potential areas for improvement in PRO research, including the need to include comprehensive PRO information in study protocols and prioritise the collection of PROs to encourage complete data and publication of results. Future research could also look in more depth at how missingness relates to specific PROMs and used to guide selection of instruments in trial design.

## Conclusion

Missing data may impact the publication of PROs. The completeness of SPIRIT-PRO checklist items in the study protocol and the use of PROs as primary endpoints may improve data completeness. This preliminary evidence suggests a future larger review investigating the relationship between PRO data missingness and publication is warranted.

## Supplementary Information

**Additional file 1: Table S1**. Protocol data extraction table.

**Additional file 2: Table S2**. Publication data extraction table.

## Data Availability

The dataset analysed during the current study are held in the Royal Marsden Data Warehouse and are available from the corresponding author on reasonable request.

## Abbreviations

PRO: Patient-reported outcome
PROM: Patient reported outcome measure
CTIMP: Clinical trials of investigative medicinal product
PREM: Patient-reported experience measure
CI: Chief investigator
EORTC: European Organisation for Research and Treatment of Cancer
QLQ-C30: Quality of Life Questionnaire-Core Module
